# A regulatory element associated to NAFLD in the promoter of *DIO1* controls LDL-C, HDL-C and triglycerides in hepatic cells

**DOI:** 10.1186/s12944-024-02029-9

**Published:** 2024-02-16

**Authors:** Casimiro Castillejo-López, José Ramón Bárcenas-Walls, Marco Cavalli, Anders Larsson, Claes Wadelius

**Affiliations:** 1 Science for Life Laboratory, Department of Immunology, Genetics and Pathology, Uppsala University, 751 08 Uppsala, Sweden Box 815, Husargatan 3, BMC,; 2 Department of Medical Sciences, Clinical Chemistry, Uppsala University Hospital, 751 85 Uppsala, Sweden

**Keywords:** Allele-specific SNPs, Functional variants, Genome editing, Differential gene expression, Phenotypic changes

## Abstract

**Background:**

Genome-wide association studies (GWAS) have identified genetic variants linked to fat metabolism and related traits, but rarely pinpoint causative variants. This limitation arises from GWAS not considering functional implications of noncoding variants that can affect transcription factor binding and potentially regulate gene expression. The aim of this study is to investigate a candidate noncoding functional variant within a genetic locus flagged by a GWAS SNP associated with non-alcoholic fatty liver disease (NAFLD), a condition characterized by liver fat accumulation in non-alcohol consumers.

**Methods:**

CRISPR-Cas9 gene editing in HepG2 cells was used to modify the regulatory element containing the candidate functional variant linked to NAFLD. Global gene expression in mutant cells was assessed through RT-qPCR and targeted transcriptomics. A phenotypic assay measured lipid droplet accumulation in the CRISPR-Cas9 mutants.

**Results:**

The candidate functional variant, rs2294510, closely linked to the NAFLD-associated GWAS SNP rs11206226, resided in a regulatory element within the DIO1 gene's promoter region. Altering this element resulted in changes in transcription factor binding sites and differential expression of candidate target genes like *DIO1*, *TMEM59*, *DHCR24*, and *LDLRAD1*, potentially influencing the NAFLD phenotype. Mutant HepG2 cells exhibited increased lipid accumulation, a hallmark of NAFLD, along with reduced LDL-C, HDL-C and elevated triglycerides.

**Conclusions:**

This comprehensive approach, that combines genome editing, transcriptomics, and phenotypic assays identified the *DIO1* promoter region as a potential enhancer. Its activity could regulate multiple genes involved in the NAFLD phenotype or contribute to defining a polygenic risk score for enhanced risk assessment in NAFLD patients.

**Supplementary Information:**

The online version contains supplementary material available at 10.1186/s12944-024-02029-9.

## Background

Genome-wide association studies (GWAS) have successfully identified genetic variants associated to fat metabolism and its related traits [1]. Well-known examples are a variant associated to myocardial infarction and low density lipoprotein cholesterol which modifies the expression of *SORT1* and alters lipid plasma levels [2]; the *FTO* allele associated to obesity, where a SNP disrupts the repressor binding which activates genes hundreds of kilobases away shifting the adipocyte differentiation from energy dissipating to energy-storing status [3]; a variant which has been associated to multiple vascular diseases and regulates the expression of *ET-1* [4]. Other successful studies have been reported in which an enhancer controls adipocyte differentiation [5].

However, very seldom GWAS are able to identify real causative variants, a limitation inherent to the study design that do not account for the functional implications of noncoding variants but instead facilitates the identification of the associated region(s) [6]. A different approach prioritizes polymorphisms in candidate loci; genotyping can indicate candidate causal variants to be further validated experimentally [7].

It has been shown that it is possible to harvest the information from signals in DNA enriched by chromatin immunoprecipitation (ChIP) differing between alleles to pinpoint allele specific SNPs (AS-SNPs). Heterozygous AS-SNPs are expected to alter transcription factors (TFs) binding and are candidate functional variants regulating gene expression [8]. AS-SNPs in high linkage disequilibrium (LD) with GWAS SNPs may therefore represent the functional drivers of the associated traits or disease. With this approach a collection of AS-SNPs in the hepatocellular carcinoma cell line HepG2 [9] and, most recently, in human liver tissue have been reported [10].

This study focuses on a locus defined by an AS-SNP which is in LD with a GWAS SNP associated with non-alcoholic fatty liver disease (NAFLD). NAFLD is a term that encompass a series of conditions characterized by a build-up of fat in liver of people that do not consume excessive alcohol. Established conditions associated to NAFLD are obesity, type 2 diabetes, hypertension and dyslipidemia with high serum triglyceride (TG) levels and low serum high-density lipoprotein (HDL) levels [11].

Two independent mutations were created for a candidate regulatory element flagged by the AS-SNP using CRISPR-Cas9 gene editing technology in HepG2 cells. Targeted transcriptomics was performed for the CRISPR-Cas9 modified cells followed by differential gene expression analysis and pathway analysis. Finally, phenotypic changes in the mutated cells were evaluated by automatic measuring fat accumulation in form of lipid droplets and the cellular levels of other lipid metabolites.

Overall, this study presents a multi-layered strategy to advance the understanding of molecular mechanisms behind complex diseases such as NAFLD. The integration of genomics, transcriptomics and phenotypic changes measurements can lead to new insights on cellular fat metabolism in the context of complex diseases.

## Methods

### Cell cultures

The human hepatocarcinoma cell line HepG2 was cultured in RPMI-1640 medium (SigmaAldrich) supplemented with 10% foetal bovine serum (FBS), penicillin (100 units/ml), streptomycin (100 µg/ml) and L-Glutamine (2 mM). The cells were incubated at 37 °C with 5% CO_2_.

### CRISPR-Cas9 guides design and selection

Single guide RNAs (sgRNA) were designed using the online tool at: www.broadinstitute.org/gpp/public/analysis-tools/sgrna-design and cloned into the BsmBI site of the lentiCRISPRv2 lentiviral vector [12]. Two sgRNA were designed to indepently target the sequence adjacent to the rs2294510 AS-SNPs upstream of the *DIO1* gene to avoid the coding sequence. In general, the guides were preferentially selected if they targeted experimental or in silico predicted transcription factors binding sites (from ENCODEdatasets) in HepG2 or liver tissue.

### Lentivirus production, purification and transduction

For lentiviral production, 4–5 × 10^5^ HEK293T cells were cultured in DMEM Glutamax medium (ThermoFisher) supplemented with 10% FBS, penicillin (100 units/ml), streptomycin (100 µg/ml) and incubated in 6-well culture plate at 37 °C, 5% CO2 overnight.

Lentiviruses containing the sgRNA, the Cas9 nuclease and puromycin N-acetyl-transferase genes were generated in HEK293T cells by co-transfection of the packaging plasmids psPAX.2 and psMD2 (Addgene). Supernatants containing lentivirus were harvested 24 h and 48 h post-transfection. Lentivirus expressing EGFP based on the pLJM1-EGFP plasmid were used as controls. The cloned sgRNAs were verified by Sanger sequencing. The transduction of the HepG2 cells was carried out overnight in OptiMEM (Gibco) containing 8 µg/ml hexadimethrine bromide (polybrene; Sigma-Aldrich) and the selection of the transduced cells was performed for 3 days with 1 µg/ml puromycin (Gibco) in growth medium.

### Assessment of CRISPR-Cas9 editing efficiency

Cells resistant to puromycin were propagated and an aliquot from each transduction, corresponding to ∼1 × 10^5^ cells, was withdrawn for genomic DNA (gDNA) extraction using phenol–chloroform method. For gDNA isolation from isogenic mutated clones, GeneJET genomic DNA purification kit (ThermoScientific) was used. The gDNA concentration was estimated with Nanodrop. PCR amplification of the target sequence was performed with 10–30 ng gDNA using Platinum Taq polymerase (ThermoFisher) and the primers shown in Supplementary Table S1. The PCR program included two touchdown cycles with annealing temperature from 57 °C to 55 °C, and 27 cycles of denaturation, annealing to 54 °C and extension steps. The PCR products were normalized to 25 ng/µL with Sequal prep plate (ThermoFisher) and Sanger sequenced in both directions using the amplification primers. Independent transductions were assessed for genome editing efficiency using Tracking of Indels by Decomposition (TIDE) [13]. The chromatograms were analysed with the online tool at https://tide.nki.nl/. For isogenic clones the serial dilution array method was used based on Corning protocol Rev02, from John A. Ryan in 96-well plate. Isolated mutant clones were verified by Sanger sequencing.

### Cellular lipid quantification assay

For phenotypic assays of lipid accumulation, HepG2 cells were grown in T-25 cell culture flasks and harvest until ≈ 70% of confluency. The cells were detached with 0.25% trypsin until a single cell suspension or small cell clusters were observed by inverted microscope. The cells were counted with EVE automatic cell counter (NanoEntek), further diluted to 5 × 10^5^ cells/ml and counted again to dilute them to a 1 × 10^5^cells/ml suspension. Later 10^4^ cells were seeded in 96-well black plates (Corning CellBIND Surface) with multichannel micropipette, ensuring a homogeneous cell suspension with regular pipetting to obtain a good distribution of the cells across the wells. Intracellular lipid accumulation was estimated by quantification of cellular lipid droplets that are composed of neutral lipids, primarily triglycerides [14, 15]. The effects on lipid accumulation of high glucose and fatty acid supplementation were tested. The supplementation of fatty acid (sodium oleate)/albumin complexes were prepared as described elsewhere [16].

The HepG2 cells were plated at the same density in DMEM-glucose (5.5 mM, termed normal glucose) or DMEM-glucose (30 mM, termed high glucose) for 24 h before supplementation with RPMI complete medium supplemented with different concentrations (0, 40, 120, 360 µM) of oleic acid (OA) or vehicle control (BSA).

The fluorescent neutral lipid dye, Bodipy 493/503 (4, 4-Difluoro-1,3,5,7,8-Pentamethyl-4-Bora-3a,4a-Diaza-s-Indacene; Molecular Probes), was used to measure the accumulation of lipid droplets. A stock solution of Bodipy 493/503 was prepared in ethanol at 1 mg/mL. Cells were treated with 0.5 μg/mL Bodipy 493/503 and 3.3 μM Hoechst 33,342 in PBS and imaged with the EVOS FL Auto Imaging System on the GFP and DAPI channels. Images were analysed with a custom Cell-Profiler pipeline that quantified the mean intensity of Bodipy per cell [15].

### Biochemical analysis of bulk cells

Culture medium was changed when cells in a T25 bottle reached 70–80% of confluence. After 24 h the cultures were treated with vehicle control or 120 μM oleic acid. The cells were harvested 24 h later by trypsinization. The cellular pellets were syringe-homogenized in 25 μl of 1% Triton in PBS and the homogenized diluted with 125 μl PBS and centrifuged at 3500 rpm for 5 min at 4 °C. The supernatant was stored at -80 °C and used for biochemical analysis and protein quantification. LDL-C, HDL-C, triglyceride, total cholesterol and glucose levels were quantified using a Mindray BS-380 analyzer (Mindray Medical International, Shenzhen, China) using direct LDL-C (1E31), HDL-C (3K33), triglyceride (7D74), cholesterol (7D62), and glucose (3L82) reagents from Abott Laboratories (Abott Park, IL, USA). Total protein of the homogenizes was used for normalization and the concentration was determined using the bicinchoninic acid (BCA) assay (Thermo Fisher).

### RNA isolation, reverse transcription and quantitative real time PCR (RT-qPCR)

Non treated HepG2 cells seeded in 12-well plates were preserved on RNAlater (SigmaAldrich) at -20 °C*.* Total RNA was isolated with RNeasy Mini kit (Qiagen) and quantified by Nanodrop spectrophotometer (ThermoScientific). Later cDNA synthesis was performed with SuperScript IV reverse trancriptase (Invitrogen) using random hexamers. qPCR reaction was performed with PowerUP Sybr Green Dye (Applied Biosystems) in a Mx3000 Pro (Stratagene). Fold induction values were calculated according to ∆∆Ct efficiency corrected mathematical model [17]. The values were normalized to TATA-Binding protein (*TBP*) expression and relative to wildtype HepG2. Primers used in qPCR are listed in Supplementary Table S2. Every reaction was analyzed at least in qPCR technical duplicates and four biological replicates were used for every mutant.

### Targeted transcriptome sequencing and data analysis.

Biological duplicates from the heterogeneous population of CRISPR-Cas9 edited HepG2 cells, isogenic clones and wildtype HepG2 were chosen for analysis. Libraries were automatically prepared with Ion Chef Kit and using Ion Ampliseq transcriptome human gene expression panel (Ampliseq panel, ThermoFisherScientific). The libraries were sequenced by the Ion S5-XL system (Ion Torrent, ThermoFisherScientific) with 150 bp read length. Differential gene expression analysis was performed with DESeq2 package [18]. Pathway analyses were performed using Enrichr [19].

### Evaluation of enhancer activity

Enhancer activity was examined by luciferase reporter gene assays in HepG2 cell line as previously described [5]. Briefly, genomic fragments of different sizes of the 5’ region of *DIO1* (324, 232 and 102 bp) were amplified from genomic DNA and cloned into the KpnI and BglII sites of the pGL4.10 firefly luciferase reporter (Promega). The minimal promoter of pGL4.26 (Promega) was inserted upstream of the luciferase gene. As negative control an empty vector with only the minimal promoter was used. As positive control we used a human 411-bp enhancer from chromosome 12 that increases luciferase expression in many cells lines [9].

Co-transfections were performed in 48-well plate at 80% confluence using Lipofectamine 3000 (Life Technologies) with 150 ng of reporter construct and 10 ng Renilla luciferase reporter vector to normalize the transfection efficiency. Cells were lysed 24 h after transfection, and ratios between Firefly luciferase and Renilla luciferase activity were measured with a dual luciferase assay (Promega) using a Varioskan Lux Microplate Reader (Thermo).

### Prediction analysis of mutagenesis of regulatory sites/factor

For the predictions of putative gain or loss of transcription binding sites due to genome editing, the sequences were analyzed with PROMO (v3.0.2) using search sites options considering human sites and factors with default options for maximum matrix disssimilarity rate. The sequences for title of 50 nts with the mutations in the middle were analized. Finally, the sTRAP tool [20], using titles of 50 nts, with transfac metazoans matrix file and chordate conserved elements as background model, was used *to analyze potential TF binding sites affected by the mutations.*

### Statistical analysis

Statistical differences were determined by Student t-test for RT-qPCR, bioimaging and biochemical assays. Statistical significance are displayed as * *P* < 0.05 < ** *P* < 0.01. *** *P* < 0.001. For DESeq2 analysis, pvalues were determined by Wald test. Adjusted p-values (False Discovery Rate) were calculated with Benjamini–Hochberg method, applied for multiple testing correction.

## Results

### Genomic landscape of a candidate regulatory region linked to NAFLD

Allele Specific SNPs (AS-SNPs) are identified from the enrichment bias of an allele at heterozygous positions from ChIP-Seq reads. The imbalance of the fraction of the reads is indicative of functional effects from a particular allele on local chromatin state [21] and can help to define candidate regulatory regions where genetic variation contributes to phenotype, a common gap not solved alone from GWAS.

This study focuses on the AS-SNP rs2294510, previously identified in HepG2 [9]. It is located in the first exon of the *DIO1* gene that codes for the enzyme iodothyronine deiodinase. In humans, *DIO1* is mainly expressed in thyroid, liver and kidney [22]. rs2294510 introduces a synonymous variant, with the alternate allele being present in 10% of the population (C/T). The AS-SNP was detected from the enrichment bias of the reference C allele from ChIP-Seq of POLR2A in HepG2 [9]. In detail, the rs2294510-C allele showed a significant higher detection of POLR2A ChIP-seq reads suggesting a higher expression of *DIO1.*

rs2294510 is in high linkage disequilibrium (LD, r^2^ = 0.96 and D' = 1) with rs11206226 reported in a GWAS for NAFLD in a japanese population [23]. Morevover, the rs2294510-C allele and the risk allele of the GWAS SNP rs11206226-A are correlated and on the same haplotype in the japanese and other populations [24]. rs2294510 is also an eQTL for the expression of the gene *YIPF1* in testis [25], and interestingly higher expression of this protein was reported as a poor prognosis marker in liver cancer [26].

**Fig. 1:**
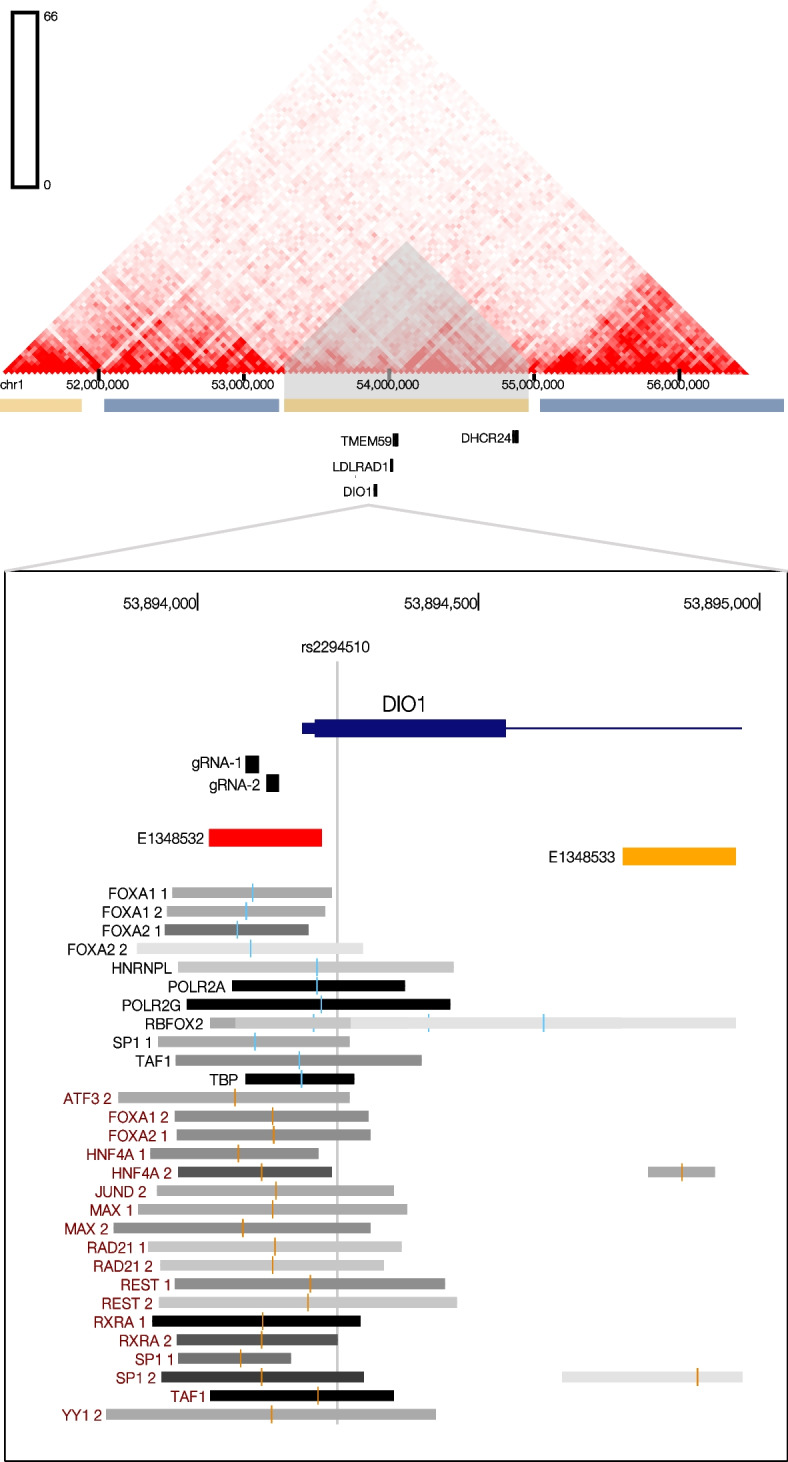
Genomic landscape of the 5’ flanking region of DIO1 gene. From the top: HiC data from Liver_STL011 in 3D Genome browser (http://3dgenome.fsm.northwestern.edu/). The AS-SNP rs2294510 is marked by a vertical line. The two guide RNAs (gRNA-1 and gRNA-2) used in the study. **A** promoter (red) and enhancer (yellow) candidate cis regulatory elements (cCREs) and transcription factors binding sites from ChIP-seq experiments in HepG2 cells (black annotations) or liver (magenta annotation) defined in ENCODE3

The ~ 2 kb genomic region surrounding rs2294510 harbors two distinct candidate cis regulatory elements (cCREs) defined in ENCODE [27]: E1548533 and E1548532, an enhancer and a promoter element respectively (Fig. 1). Proceeding from the hypothesis that the AS-SNP rs2294510 is flagging a genomic region that is more likely to explain the association to the NAFLD phenotype observed in the original GWAS, this study focused on the non-coding region upstream of rs2294510 and of the *DIO1* transcription start site (TSS).

The 5’ flanking region of *DIO1* (from now on referred to as UpsDIO1) presents signals for multiple predicted (JASPAR CORE 2022 [28]) and experimentally validated binding sites of transcription factors (TFs) such as FOXA1, FOXA2, SP1, TAF1, TBP, HNRNPL, POLR2A, RBFOX2, ATF3, HNF4A, JUND, MAX, RAD21, REST and YY1 defined by ENCODE in HepG2 and liver [27] (Fig. 1).

UpsDIO1 features a dual role as both *DIO1* proximal promoter and enhancer for distal genes. The enhancer activity of UpsDIO1 was also confirmed by luciferase reporter assays performed in HepG2 cells (Supplementary Figure S1).

Moreover, the candidate regulatory region UpsDIO1 is localized inside a Topological Associated Domain (TAD) in HepG2 cells. This ~ 1 Mb TAD harbors *DIO1* and several other genes with relevant metabolic functions including Transmembrane protein 59 (*TMEM59)*, 24-dehydrocholesterol reductase (*DHCR24*) and low-density lipoprotein receptors* (LDLRAD1)* [29].

### CRISPR-Cas9 genome editing of UpsDIO1

CRISPR-Cas9 genome editing in HepG2 was performed by designing two independent single guide RNAs (gRNAs) targeting UpsDIO1 (gRNA-1 and gRNA-2). Both gRNAs were designed to avoid direct modifications to the transcription initiation sites and translation start points of *DIO1* (Fig. 1). In principle, the genetic perturbation by CRISPR-Cas9 indels of noncoding DNA at potential regulatory elements could help to draw causal links between sequence and cellular functions [30]. The rate of Indel mutation measured by Tracking of Indels by Decomposition (TIDE) [13] was 90% for gRNA-1 and 88% for gRNA-2.

Figure 1 shows how both gRNA-1 and gRNA-2 were designed to target the region of UpsDIO1 rich in transcription factor binding sites (TFBSs). For example, gRNA-2 hits a predicted binding site for ZNF24 [28], described as trans-repressor in mammalian cells [31], and for KLF5 previously reported to play a key a role in adipocyte differentiation [32]. The gRNA-1 hits a predicted binding site for ZNF282 and RARA::RXRA and also targets the functional defined Thyroid hormone Response Element 1 (TRE1) which has previously been shown to act both as a thyroid response element and a retinoic acid response element [33]. The majority of the recovered alleles using the gRNA-1 were deletions ranging from 4 to 7 nucleotides and for gRNA-2 single nucleotide insertions (Supplementary Figure S2).

The predictions of putative gain or loss of TFBS due to genome editing was performed using different tools. The gRNA-1 deletions reduced the affinity to MYOD1 as well as removing RXRA, ESR1, RARA and JUN binding sites while creating a TFII-I and c-Ets-1 binding sites. gRNA-2 created an extension of the binding site of PAX5, a new binding site to the transcription regulator HIC1, and higher affinity for HNF4A and GATA1 for the mutant allele [34]. Furthermore, gRNA-2 created a new binding sites for FOXP3 and RXRA [35]. Overall, both gRNAs targeting UpsDIO1 showed a high potential of altering a regulatory element important for transcription or repressor factors binding.

### CRISPR-Cas9 genome editing of UpsDIO1 impacts global transcriptional deregulation that mimics NAFLD affected pathways

Regulatory elements commonly exert their function in the proximal regions within TADs, enabling intradomain enhancer promoter contacts [36, 37]. Based on these premises, indels introduced by genome editing might generate a change in gene expression of *cis*-regulated genes. To explore this possibility the differential expression of candidate target genes regulated in *cis* from UpsDIO1 in both mutants was analyzed, looking for gene(s) potentially driving the association to the NAFLD phenotype.

The expression level of *DIO1*, *TMEM59*, *DHCR24* and *LDLRAD1* was analyzed using RT-qPCR on non-isogenic HepG2 mutants by gRNA-1 and gRNA-2. These target genes were selected since (i) their transcripts or proteins are known of being expressed in human liver tissue or HepG2, (ii) have metabolic function or are implicated in lipid/cholesterol processes, and (iii) are located in the same TAD (Fig. 1).

**Fig. 2:**
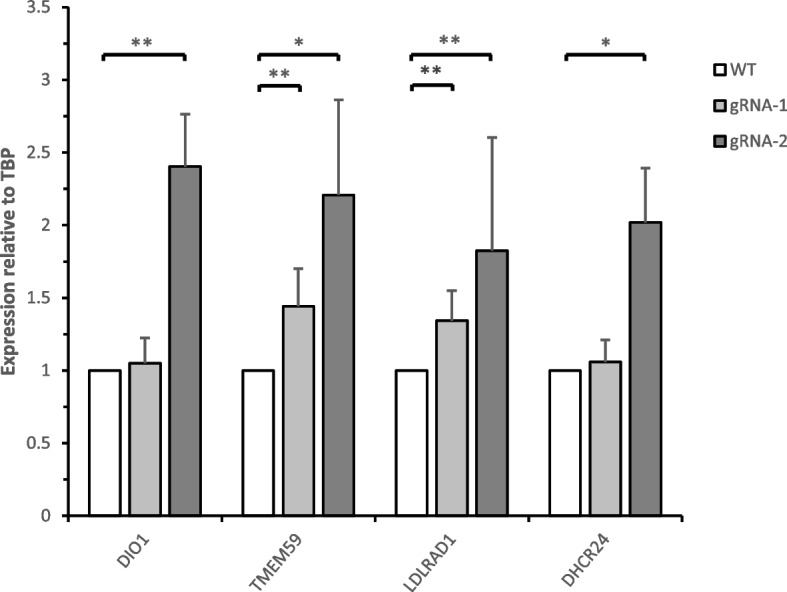
Relative gene expression analysis of CRISPR-Cas9 gRNA-1 and gRNA-2 mutants for candidate genes. Gene expression levels were analyzed with RT-qPCR, relative to TATA binding protein (TBP) and wildtype HepG2 (WT) as control. The values are given as means and standard deviations of the results from four biological replicates, *P* values derived from unpaired T-Test. *P =  < 0.05,**P =  < 0.01

The results indicated that gRNA-2 mutants led to a general overexpression of these metabolic relevant genes (Fig. 2) while gRNA-1 showed significant overexpression for only *LDLRAD1* and *TMEM59*.

Next, targeted transcriptomics was performed to study the global gene expression of isogenic cultures isolated from single mutant cells compared to wild type (WT) HepG2 cells. Since gRNA-1 and gRNA-2 mutant clones clustered in opposite directions from WT in PCA (Supplementary Figure S3) the comparisons of isogenic mutants from the same gRNA against WT HepG2 were considered separately.

Analysis by DESeq2 [18] identified 686 differentially expressed genes (DEGs), 399 dowregulated, 287 upregulated for the gRNA-1 mutants and 164 DEGs (69 dowregulated, 95 upregulated) for gRNA-2 mutants (Supplementary Table S3).

Gene Ontology (GO) terms enrichment analysis of biological processes for DEGs in gRNA-1 mutants revealed many cholesterol and lipid metabolic processes as top hits, consistent with the NAFLD framework. Pathway enrichment analysis considering only the top 20 DEGs (Elsevier Pathway Collection) showed gluconeogenesis impairment in NAFLD as top hit. Furthermore, enrichment for terms in the Jensen diseases dataset showed fatty liver disease among the top hits. GO molecular functions, pathways and diseases analysis enrichment results [19] are summarised in Supplementary Table S4.

DEGs in gRNA-2 mutants showed a GO term enrichment for estrogen hydroxylase activity, insulin-like growth factor II binding, fatty-acid-CoA synthase activity, retinoid binding, lipoprotein particle binding and LDL particle binding. When considering only the top 20 DEGs there was enrichment of molecular function for retinoid binding, P-type sodium transporter activity, P-type sodium:potassium-exchanging transporter activity, sodium ion binding and insulin-like growth factor II binding.

### CRISPR-Cas9 genome editing of UpsDIO1 increases lipid accumulation in HepG2 cells

**Fig. 3:**
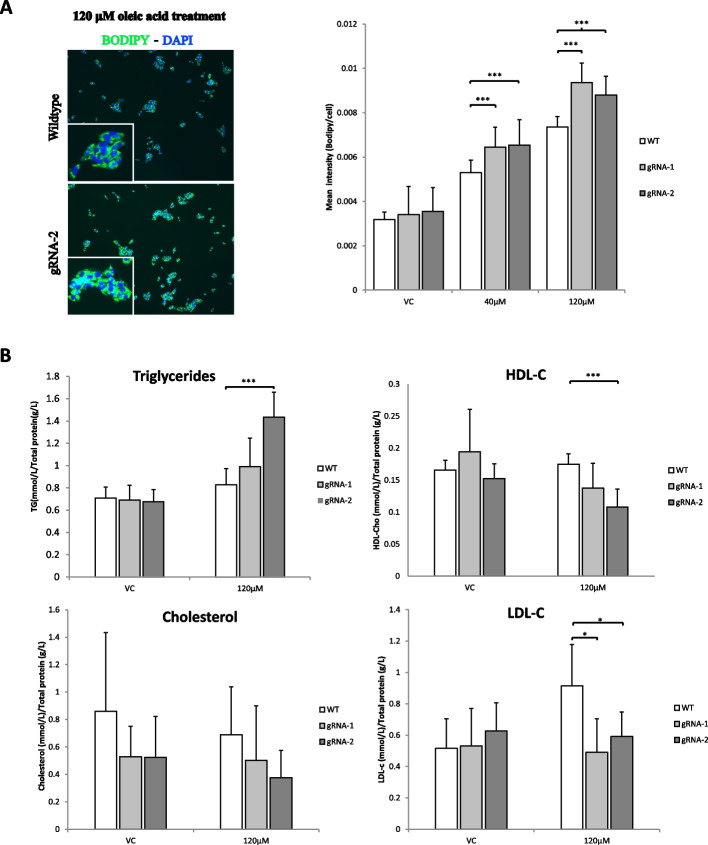
Quantification of neutral lipids and biochemical assays of UpsDIO1 CRISPR-Cas9 HepG2 mutants. **A** Representative pictures of merged channels for Hoechst and Bodipy dyes of HepG2 cells treated with 120 µM oleic acid (OA). Pictures were taken at 10X objective magnification. Bodipy quantification of UpsDIO1 mutants (gRNA-1 and gRNA-2) show differential accumulation of neutral lipids at 40 and 120 µM OA in comparison to wildtype (WT) HepG2. In total 9 pictures were taken from each culture (*n* = 3) and the mean and standard deviation of the mean (SEM) per cell is presented. **B** Biochemical quantification of culture cell extracts after treatment with 120 µM OA for triglycerides, total cholesterol, LDL-C and HDL-C. For Intracellular lipid accumulation and biochemical quantification *P*-value derived from unpaired T-Test. *P =  < 0.05,**P =  < 0.01, ***P =  < 0.001

GO, pathway and disease analysis pointed to an overall deregulation of lipid and metabolic processes as a result of genome editing of UpsDIO1 and consequent alteration of gene(s) expression that could contribute to the NAFLD pathobiology. Next, a phenotypic assay was used to measure lipid droplet accumulation in the CRISPR-Cas9 non-isogenic mutants in response to lipid and glucose overloading. The heterogeneous population of CRISPR-Cas9 edited HepG2 cells were challenged with oleic acid and high glucose supplementation treatments in cell culture. The intracellular fat accumulation in the form of lipid droplets was then characterized by fluorescent staining with the neutral lipid stain Bodipy 493/503. A lipid supplementation titration showed that oleic acid at 360 µM induced cellular toxicity (data not shown) and at 120 µM the greatest differences between cultures without compromising viability was obtained. After treatment with 40 µM and 120 µM of oleic acid it was observed a consistent increase of neutral lipid staining with mutations generated at UpsDIO1 (Fig. 3).

Cells edited using both gRNA-1 and gRNA-2 showed higher increase of lipid accumulation than the wild type cultures when challenged with oleic acid. After treatment with high glucose medium an increased trend in accumulation of intracellular lipids was observed, however no significant difference was registered between mutants against wild type cells, treated with vehicle control (VC), high or low glucose medium (Supplementary Figure S4).

To validate the above results, a biochemical assay (see Methods) was used to quantify triglycerides and other relevant metabolites such as glucose, total cholesterol, LDL-C and HDL-C. With this independent method it was found that the concentration of triglycerides normalized by total protein was higher in gRNA-2 cells compared with WT cells after oleic acid treatment. Cells edited using gRNA-1 also showed an increase in triglycerides however it did not reach a significant statistical level (Fig. 3). gRNA-2 mutant cells also showed a significant reduction of HDL-C under oleic acid supplementation (Fig. 3). For LDL-C a statistically significant reduction was found for both gRNAs. For cholesterol a reduction trend was observed for both mutant cells, however they did not reach statistical significance.

## Discussion

This study investigated the candidate genomic regulatory element flagged by AS-SNP rs2294510 as a potential driver of the association of GWAS SNP rs11206226 to NAFLD. CRISPR-Cas9 genome editing was used to perturb UpsDIO1, the adjacent promoter region of the *DIO1* gene, rich in TFBSs and study the downstream effects on gene expression and cell phenotype.

Overall, an upregulation of *DIO1*, *TMEM59*, *DHC24* and *LDLRAD1* expression was observed. The effect of genome editing effect of single gRNAs might be diluted as targeting by Cas9 results in different hetero-homozygote mutations in different cells [30]. Further selecting two isogenic clones for each gRNA, global transcriptomics and differential gene expression analysis were performed to discern whether the limits of disregulation were TAD restricted or the mutants lead to an overall change in gene expression.

Isogenic mutants from gRNA-1 showed a downregulation of *DIO1* consistent with the disruption of the TRE1 binding site in UpsDIO1 that breaks the positive feedback loop with triiodothyronine (T3) increasing the expression of *DIO1* [33, 38, 39], and an important region for basal activity and LXRα activation of *DIO1* [40]. Remarkably, from ChIP-seq studies of mice liver tissue, it was found that transcriptional induction of *DIO1* is associated to de novo and T3 induced chromatin remodelling of regulatory sites near *DIO1* promoter. *DIO1* belongs to a class of genes where thyroid hormone receptor (TR) binding is ligand dependent, where T3 leads to a dramatic remodelling of TR binding sites, and an increased recruitment of CBP coactivator [41]. The overexpression of *DIO1* observed in bulk from gRNA-2 mutants, where the TRE1 site was not affected, could instead be related to the disruption of repressive TFBS.

Several other DEGs observed in gRNA-1 isogenic mutants were previously reported in a differential gene expression analysis between mild and advanced NAFLD in liver [42]. The genes *AKR1B10*, *CHI3L1*, *KRT23* were upregulated whereas *GNMT* and *FITM1* were downregulated in agreement with the observation in this study. The downregulation of the gene glycine N-methyltransferase (*GNMT*), mimics the loss of GNMT in liver oxidative metabolism which promotes liver steatosis. GNMT has been found absent or lowly expressed in patients with NAFLD or HCC [43, 44].

Interestingly, it was also observed in gRNA-1 mutants a downregulation of patatin-like phospholipase domain-containing protein 3 (*PNPLA3*) a protein with both catabolic lipase activity and anabolic lipogenic activity. A nonsynonymous variant in *PNPLA3* has been associated with steatosis, steatohepatitis and hepatic fibrosis and its deregulation can lead to increased hepatocellular triglyceride accumulation, impairing lipid remodeling and turnover [45] and development of NAFLD [46].

Genetic variants in *GKPR*, glucokinase regulatory protein were associated to steatosis and NAFLD [47] and the gene was also downregulated in the differential expression analysis of this study. Other well-known genetic risk factor for NAFLD are variants in genes *PNPLA3*, *GKPR*, *TM6SF2*, *MBOAT7* and *HSD17B13* which have also been tested towards the definition of a polygenic risk score (PRS) for NAFLD [48].

Taken together, GO and pathway analysis performed on the collections of gRNA-1 and -2 specific DEGs revealed that genome editing of the UpsDIO1 region at experimental validated and in silico predicted TFBS, lead to a global dysregulation of genes that include important hallmarks of NAFLD pathobiology.

The phenotypic assays demonstrated that disrupting the regulatory element UpsDIO1 by CRISPR-Cas9 lead to higher accumulation of lipids in mutant HepG2 cells after lipid overloading as an in vitro model of steatosis (Fig. 3). Interestingly, similar treatment with oleic acid and palmitic acids to mouse liver cells observed an increase of *DIO1* mRNA expression [49] suggesting the disruption of a potential active and/or repressive TFBS might dysregulate *DIO1* expression or other cis-regulated genes required for fatty acid removal or oxidation.

In addition, liver-specific knockdown of *DIO1* in mice, showed an increase of triglycerides and cholesterol; and with lipid overloading of mice liver cells an increase of fat content compared to control cells [49]. DIO1 missense variants has been found to reduce *DIO1* activity, and caused elevated reverse iodothyronine (rT3) and rT3/T3 ratio in humans [50] and instead alleles that cause higher deiodinase I activity increase of circulating free triiodothyroxine to thyroxine (T3/T4) ratio and free T3 [51]. Future studies will be required to determine if polymorphisms in the UpsDIO1 region act through missregulation of *DIO1* alone, or by other contributing genes through UpsDIO1 as an enhancer with cis- regulatory activity. The findings from this study suggest a novel regulatory role for UpsDIO1 in NAFLD, where noncoding mutations at this regulatory element increase lipid accumulation in hepatocytes, a pathological hallmark of NAFLD, and dysregulated the expression of genes involved in cholesterol and lipid metabolism processes.

### Study strengths and limitations

The strategy utilized in this study reflects an increasingly popular approach in biomedical research that apply multi-omics strategies to the study of complex diseases. The ultimate goal is to obtain a comprehensive view of biological systems underlying complex diseases by harvesting information from multiple molecular levels. The main strength of this study relies on the integration of genomics and transcriptomics that allowed investigating the effects of genomics alterations to the expression of specific genes and the alteration of phenotypes in the context of NAFLD.

However, the definition of the molecular mechanism connecting genomics alterations of UpsDIO1 to gene expression and altered lipids uptake phenotypes remains elusive. The main limitation of this study is that in silico predictions of TFBS alterations which could mediate these effects can only provide a starting point for further experimental investigations that will be required to move towards a better understanding and treatment of NAFLD.

## Conclusions

While more experimental validations are needed to unveil specific molecular mechanisms of action, the multi-layered strategy presented in this study by combining genome editing, transcriptomics and phenotypic assays flagged the promoter region of *DIO1* as a putative enhancer. Its activity could potentially regulate the expression of several genes that might participate to the NAFLD phenotype. On a clinical point of view, the findings could contribute to the definition of a polygenic risk score to improve the risk stratification of NAFLD patients, however their clinical significance needs to be studied in suitable patient cohorts.

### Supplementary Information

**Additional file 1: Supplemental Figure S1.** Evaluation of enhancer activity by Luciferase reporter assay. **Supplemental Figure S2.** Estimation of genome editing efficiency by TIDE. Indel spectrum of UpsDIO1 mutants assessed by Tracking of Indels by Decomposition (TIDE). A) Indel spectrum of gRNA-1 bulk mutant B) Indel spectrum of gRNA-2 bulk mutant. **Supplemental Figure S3.** PCA of gRNA-1, gRNA-2 mutant clones and WT HepG2 cells. **Supplemental Figure S4.** A) Biochemical quantification of culture cell extracts after treatment with 120 µM OA for glucose. B) Lipid accumulation by glucose overloading.

**Additional file 2: Supplemental Table S1. **Single guide RNA sequences before cloning in lentiCRISPRv2. **Supplemental Table S2.** Single guide RNA sequences before cloning in lentiCRISPRv2.

**Additional file 3.**

**Additional file 4.**

## Data Availability

The datasets utilized and/or analyzed during the current study are available from the corresponding author upon reasonable request.

## Abbreviations

AKR1B10: Aldo-Keto Reductase Family 1 Member B10
AP-1: Transcription Factor Subunit
AS-SNPs: Allele Specific SNPs
ATF3: Activating Transcription Factor 3
cCREs: Candidate cis-Regulatory Elements
c-Ets-1: ETS Proto-Oncogene 1 Transcription Factor
CHI3L1: Chitinase 3 Like 1
ChIP: Chromatin Immunoprecipitation
CRISPR-Cas9: Clustered Regularly Interspaced Short Palindromic Repeats associated protein 9
DEGs: Differentially Expressed Genes
DHCR24: 24-Dehydrocholesterol reductase
DIO1: Iodothyronine Deiodinase 1
eQTLs: Expression Quantitative Trait Loci
ESR1: Estrogen Receptor 1
ET-1: Endothelin 1
FITM1: Fat Storage Inducing Transmembrane Protein 1
FOXA1: Forkhead Box A1
FOXA2: Forkhead Box A2
FOXP3: Forkhead Box P3
FTO: Alpha-ketoglutarate-dependent dioxygenase
GATA1: GATA Binding Protein 1
GKPR: Glucokinase Regulatory Protein GNMT Glycine N-Methyltransferase
GO: Gene Ontology
gRNAs: Guide RNAs
GWAS: Genome-Wide Association Studies
HCC: Hepatocellular Carcinoma
HDL-C: High-density lipoprotein cholesterol
HIC1: HIC ZBTB Transcriptional Repressor 1
HNF4A: Hepatocyte Nuclear Factor 4 Alpha
HNRNPL: Heterogeneous Nuclear Ribonucleoprotein L
HSD17B13: Hydroxysteroid 17-Beta Dehydrogenase 13
JUN: Jun Proto-Oncogene
JUND: JunD Proto-Oncogene
KLF5: KLF Transcription Factor 5
KRT23: Keratin 23
LD: Linkage Disequilibrium
LDL-C: Low-density lipoprotein cholesterol
LDLRAD1: Low-density lipoprotein receptors
MAX: MYC Associated Factor X
MBOAT7: Membrane Bound O-Acyltransferase Domain Containing protein 7
MYOD1: Myogenic Differentiation 1
NAFLD: Non-Alcoholic Fatty Liver Disease
PAX5: Paired Box 5
PNPLA3: Patatin-like phospholipase domain-containing protein 3
POLR2A: RNA Polymerase II Subunit A
RAD21: RAD21 Cohesin Complex Component
RARA: Retinoic Acid Receptor Alpha
RBFOX2: RNA Binding Fox-1 Homolog 2
REST: RE1 Silencing Transcription Factor
RT-qPCR: Real-time quantitative polymerase chain reaction
RXRA: Retinoid X Receptor Alpha
SNP: Single-nucleotide polymorphism
SORT1: Sortilin 1
SP1: Sp1 Transcription Factor
TAD: Topological associated domain
TAF1: TATA-Box Binding Protein Associated Factor 1
TBP: TATA-Box Binding Protein
TFBSs: Transcription factor binding sites
TFII-I: General Transcription Factor Iii
TFs: Transcription factors
TG: Triglyceride
TIDE: Tracking of indels by decomposition
TM6SF2: Transmembrane 6 Superfamily Member 2
TMEM59: Transmembrane protein 59
TRE1: Thyroid hormone Response Element 1
TSS: Transcription start site
VC: Vehicle control
WT: Wild type
YIPF1: Yip1 Domain Family Member 1
YY1: YY1 Transcription Factor
ZNF24: Zinc Finger Protein 24
ZNF282: Zinc Finger Protein 282
