# Supplementary material for: A regulatory element associated to NAFLD in the promoter of DIO1 controls LDL-C, HDL-C and triglycerides in hepatic cells
Source: Lipids Health Dis. 2024 Feb 16;23:48. doi: 10.1186/s12944-024-02029-9 (PMC10870585; doi:10.1186/s12944-024-02029-9)
Supplement: Supplementary file 1 — Additional file 1: Supplemental Figure S1. Evaluation of enhancer activity by Luciferase reporter assay. Supplemental Figure S2. Estimation of genome editing efficiency by TIDE. Indel spectrum of UpsDIO1 mutants assessed by Tracking of Indels by Decomposition (TIDE). A) Indel spectrum of gRNA-1 bulk mutant B) Indel spectrum of gRNA-2 bulk mutant. Supplemental Figure S3. PCA of gRNA-1, gRNA-2 mutant clones and WT HepG2 cells. Supplemental Figure S4. A) Biochemical quantification of culture cell extracts after treatment with 120 µM OA for glucose. B) Lipid accumulation by glucose overloading. [file 12944_2024_2029_MOESM1_ESM.docx]

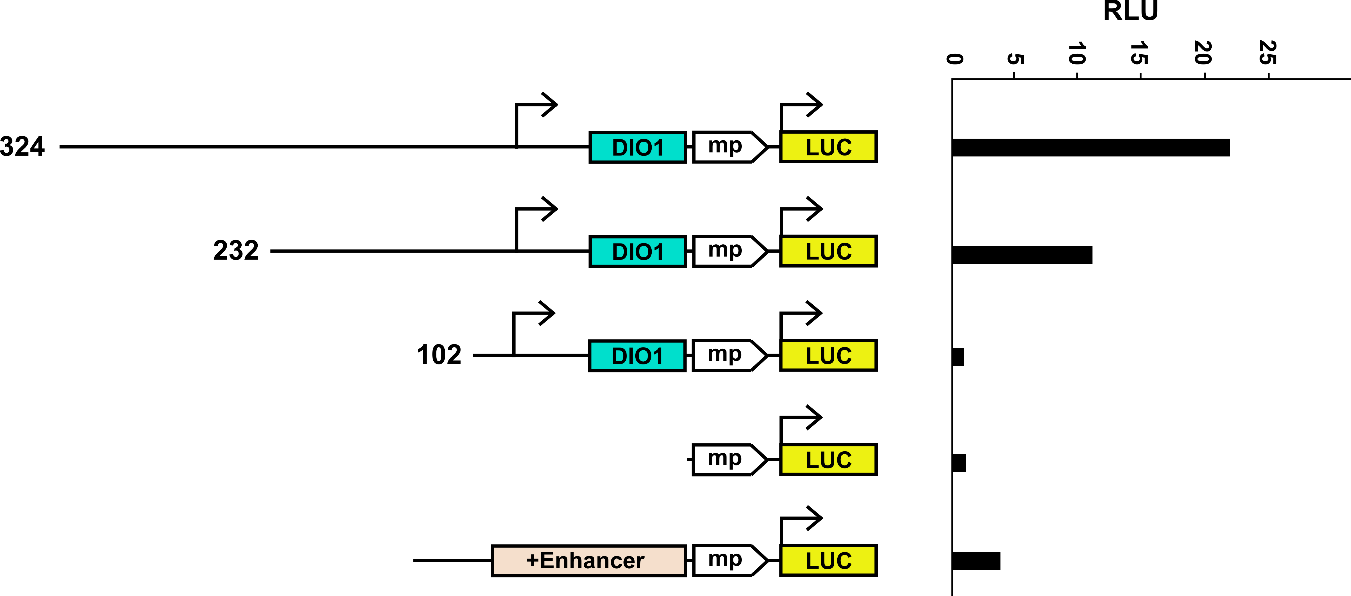


**Supplemental Figure S1**. Evaluation of enhancer activity by Luciferase reporter assay.

1. **gRNA-1- Bulk culture**Total efficiency: 97.8%. R^2^ = 0.98


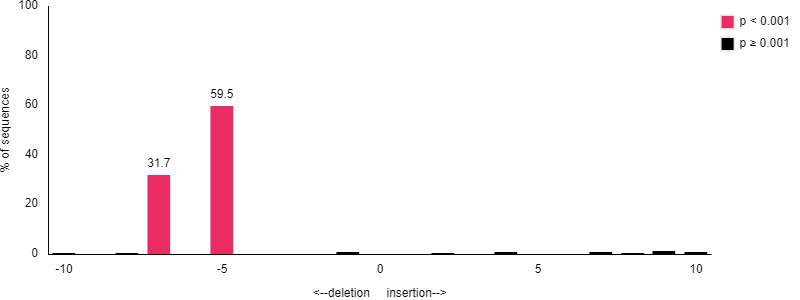


1. **gRNA-2 Bulk culture**Total efficiency: 88.5%. R^2^ = 0.99

**
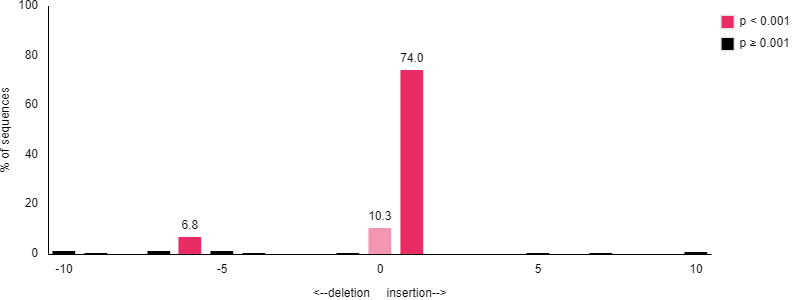
**

**Supplemental Figure S2.** Estimation of genome editing efficiency by TIDE. Indel spectrum of UpsDIO1 mutants assessed by Tracking of Indels by Decomposition (TIDE). A) Indel spectrum of gRNA-1 bulk mutant B) Indel spectrum of gRNA-2 bulk mutant.


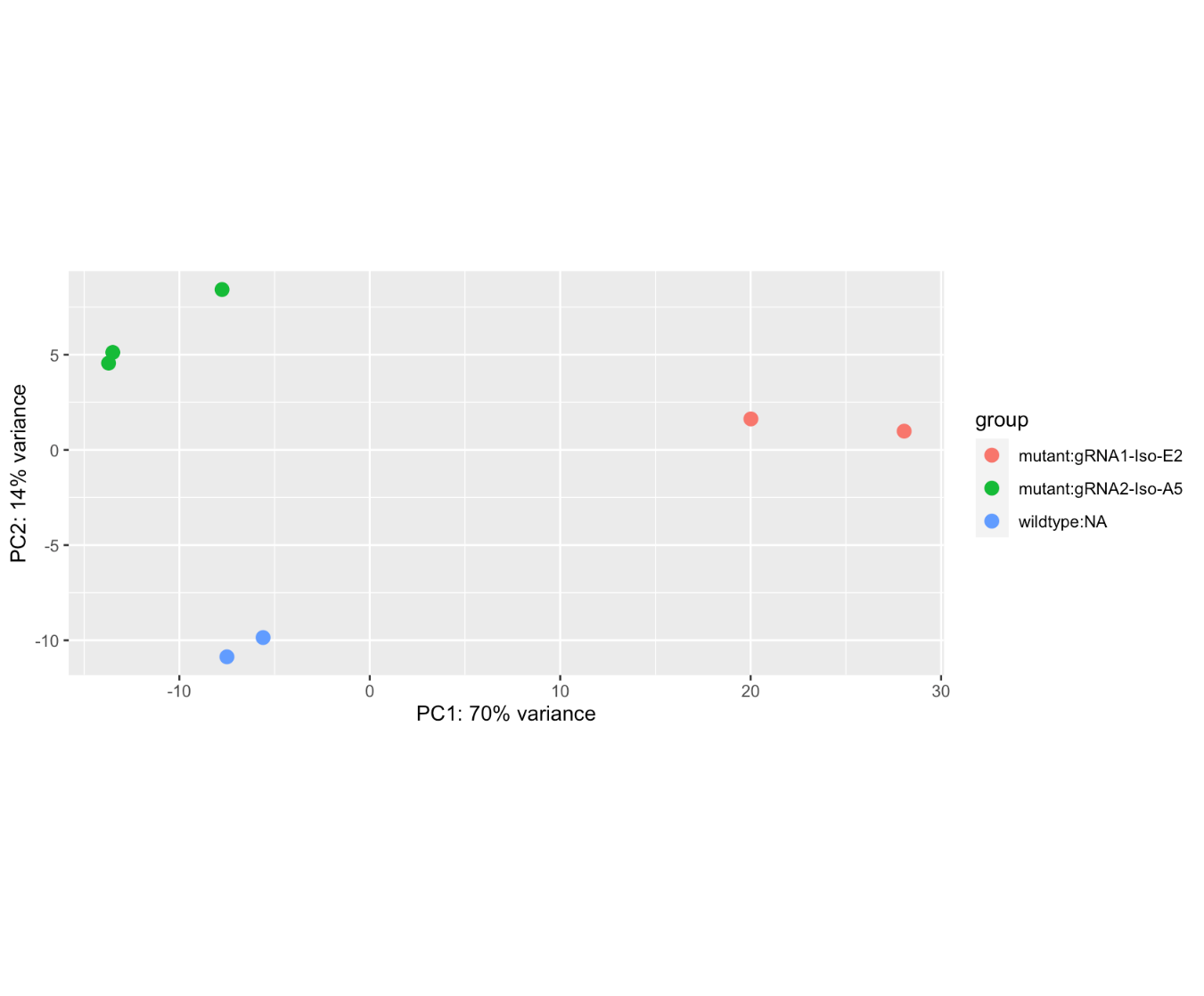


**Supplemental Figure S3**. PCA of gRNA-1, gRNA-2 mutant clones and WT HepG2 cells


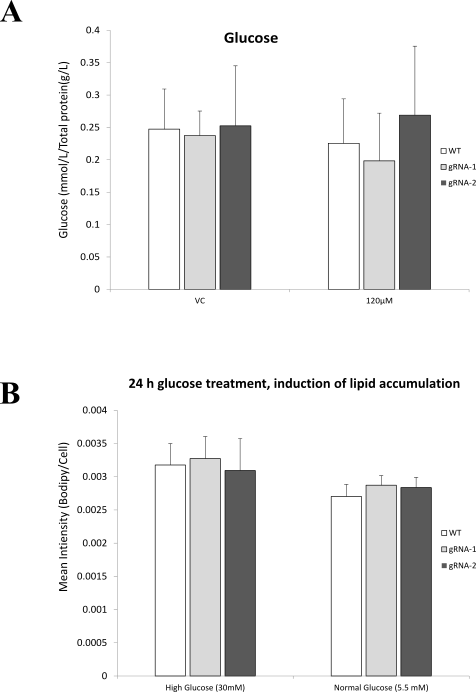


**Supplemental Figure S4.** A) Biochemical quantification of culture cell extracts after treatment with 120 µM OA for glucose. B) Lipid accumulation by glucose overloading.
