# Supplementary material for: A regulatory element associated to NAFLD in the promoter of DIO1 controls LDL-C, HDL-C and triglycerides in hepatic cells
Source: Lipids Health Dis. 2024 Feb 16;23:48. doi: 10.1186/s12944-024-02029-9 (PMC10870585; doi:10.1186/s12944-024-02029-9)
Supplement: Supplementary file 2 — Additional file 2: Supplemental Table S1. Single guide RNA sequences before cloning in lentiCRISPRv2. Supplemental Table S2. Single guide RNA sequences before cloning in lentiCRISPRv2. [file 12944_2024_2029_MOESM2_ESM.docx]

**Supplemental Table S1**. Single guide RNA sequences before cloning in lentiCRISPRv2.

| **AS-SNP** | **Guide RNA Name** | **Target sequence (5' → 3')** | **Oligo 1 (5' → 3')** | **Oligo2 (5' → 3')** |
| --- | --- | --- | --- | --- |
| **rs22941510** | gRNA-1 | TGACTCCTTCCCCTGACCCGGGG | caccgTGACTCCTTCCCCTGACCCG | aaacCGGGTCAGGGGAAGGAGTCAc |
| **rs22941510** | gRNA-2 | CCCTGCACCTTCATTCAGCTCCG | caccgCGGAGCTGAATGAAGGTGCA | aaacTGCACCTTCATTCAGCTCCGc |

**Supplemental Table S2**. Primer sequences of RT-qPCR for candidate target genes deregulated after CRISPR-Cas9 mutagenesis.

| **Target Gene/Transcript** | **Direction** | **Sequence (5' → 3')** | **Ø Tm [°C]3** | **GC%** | **Product Lenght(bp)** |
| --- | --- | --- | --- | --- | --- |
| LDLRAD1 | Forward | ATGGCGTTCGCACCTGTA | 59.34 | 55.56 | 150 |
| LDLRAD1 | Reverse | AGTTGTTAGTGCCATCACATTTTTGG | 60.96 | 38.46 | 150 |
| DIO1 | Forward | GTTGGCAGCGACTAGAGGAC | 60 | 60 | 138 |
| DIO1 | Reverse | AACTTCCAAAATTCAGCACCA | 59.6 | 38.1 | 138 |
| TMEM59 | Forward | AGAGGTTGCAGGCTGTTTTCAA | 60.95 | 45.45 | 149 |
| TMEM59 | Reverse | AGCTGATTCTGGCAACCAAGA | 59.93 | 47.62 | 149 |
| DHCR24 | Forward | GCTGCTCTACTCCCTGGATGA | 58.4 | 57.1 | 149 |
| DHCR24 | Reverse | GGCCCTCTCGGTTTGTCTT | 57.4 | 57.9 | 149 |
